# A stump appendicitis in a child: a case report

**DOI:** 10.1186/1824-7288-35-35

**Published:** 2009-11-17

**Authors:** Manef Gasmi, Fatma Fitouri, Sondes Sahli, Radhia Jemaï, Mourad Hamzaoui

**Affiliations:** 1 Department of Paediatric Surgery "A", Children Hospital, Tunis, Tunisia

## Abstract

**Background:**

Stump appendicitis is a delayed complication of appendectomy. It is rare and few cases reported in the paediatric literature. The authors report on another case in a child and focus on the diagnostic peculiarities of this entity.

**Case:**

A 9-year-old boy with previous history of open appendectomy was admitted for a right lower quadrant pain with bilious vomiting and fever. Physical examination demonstrated tenderness in the right lower quadrant and guarding over the appendectomy scar. The white blood cell count was 23.500 cells/mm^3^. Plain abdominal radiograph and ultrasonography revealed fecalith localized in the right iliac fossa. The diagnosis of stump appendicitis was advocated and confirmed at laparotomy. A gangrenous and perforated appendiceal stump was found and completely removed. The post-operative course was uneventful after 18 months follow-up period.

**Conclusion:**

Stump appendicitis is rare and should be considered in any patient with right lower quadrant pain even if there is a history of appendectomy. Complete removal of the appendix is the only mean to prevent the occurrence of this complication.

## Background

Stump appendicitis is a delayed complication of appendectomy. It is rare and few cases reported in the paediatric literature. The authors report on another case in a child and focus on the diagnostic peculiarities of this entity.

## Case

**Figure 1:**
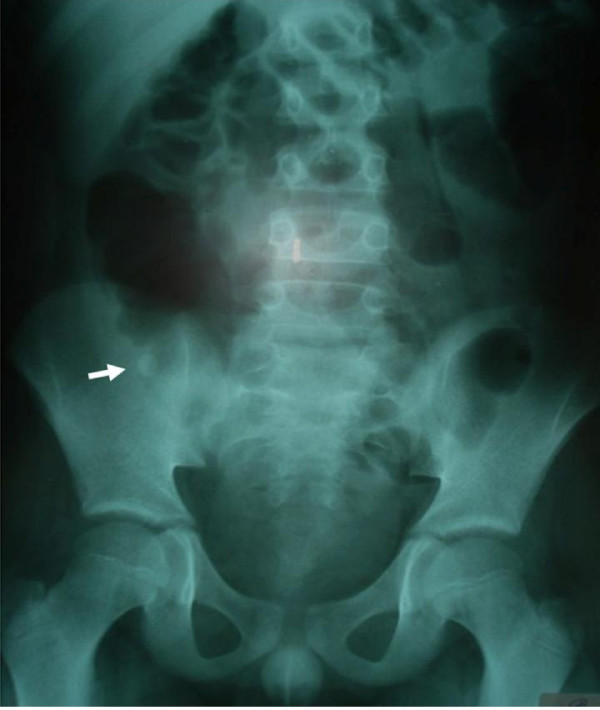
**Plain abdominal radiograph demonstrating fecalith (arrow)**.

**Figure 2:**
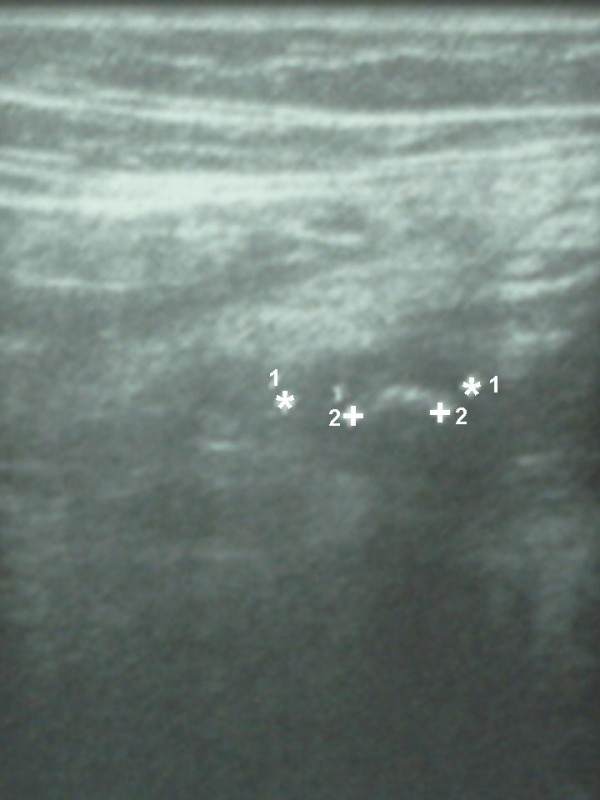
**Ultrasonography revealing fecalith (1) into appendiceal stump (2)**.

**Figure 3:**
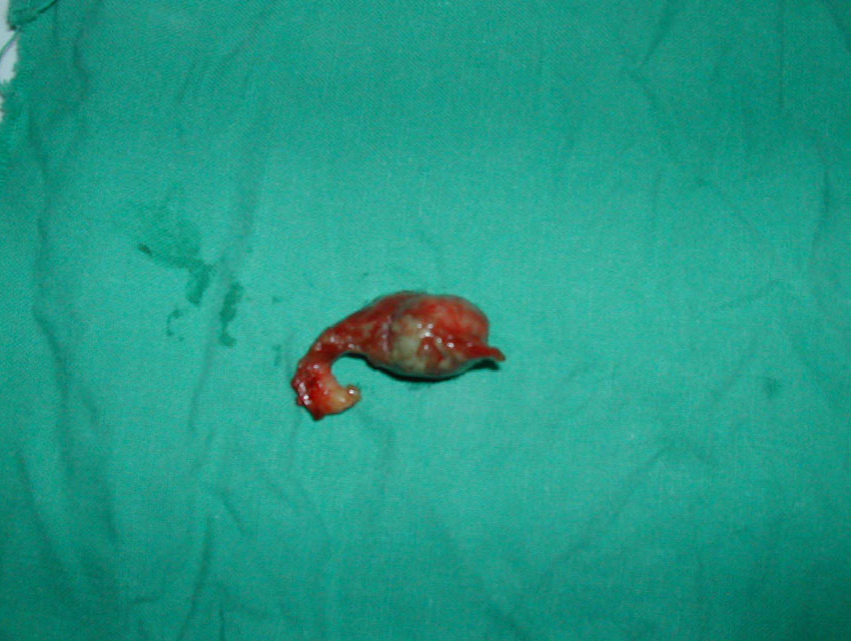
**Pathologic specimen showing gangrenous and perforated appendiceal stump**.

A 9-year-old boy was admitted with a chief complaint of right lower quadrant pain of 24 hours duration associated to bilious vomiting and fever. His surgical history reveals an open appendectomy performed three years ago. On physical examination, he was not in distress and has a fever of 39°C. Abdominal palpation demonstrated tenderness in the right lower quadrant and guarding over the appendectomy scar. The remainder of the abdomen was soft and nontender. However, no abdominal masses were appreciated and the rectal and scrotal examinations were normal. The white blood cell count was 23.500 cells/mm^3 ^with 87% neutrophils. Plain abdominal radiograph (figure 1) and ultrasonography (figure 2) revealed fecalith localized in the right iliac fossa. The diagnosis of stump appendicitis was advocated and confirmed at laparotomy. Through the same Mac Burney's approach, a gangrenous and perforated appendiceal stump of 35 mm was found and completely removed (figure 3). The post-operative course was uneventful after 18 months follow-up period.

## Discussion

Stump appendicitis is the re-inflammation of the residual appendiceal tissue after an appendectomy [1-3]. It represents a rare delayed complication of appendectomy which is unknown by most clinicians [1-7]. Its frequency is under-estimated and under-reported [4,5,7,8]. Some factors have been suggested for the development of this condition. An appendicael stump that is left too long represents the most advocated etiologic factor [1-5,7-12]. Our patient had a relatively long stump. Inadequate identification of the appendicael base, because of severe local inflammation, retrocecal or sub-serous appendix, has been also suggested [1,4,5,10]. Moreover, the incidence of this complication seems to increase until the introduction of laparoscopic approach, probably due to absence of tactile feedback [1,4,13]. The age of the patients ranges from 11 to 72 years with [1,2,4,14]. Only three cases are reported in the paediatric literature [1,2,14]. The time of onset ranges from 2 weeks to decades after appendectomy [1,2,5,8-10,14-16]. The recognition of stump appendicitis can be challenging and are often delayed, leading to serious complications [1,4,8,16]. Thus, early diagnosis is necessary and should be considered when evaluating any patient with recurrent right lower quadrant abdominal pain and a history of appendectomy [16]. Clinically, patients present with signs and symptoms similar to appendicitis or acute abdomen [6]. The presence of an appendectomy scar does not absolutely rule out the possibility of stump appendicitis. Physician should keep in mind a possible incomplete appendiceal resection to prevent delayed diagnosis and treatment. As in our case, a high clinical suspicion and the presence of fecalith may help to diagnose the disease. Ultrasonography and CT scan of the abdomen constitute the modalities of choice for confirming the diagnosis [2,4,5,9,11,14]. Laparoscopy seems to be better than conventional laparotomy. It permits to perform a global ispection of abdominal cavity and an easier adhesiolysis [10,11,14]. Treatment is based on complete removal of the appendix [2,8].

## Conclusion

Stump appendicitis is a real entity. Its diagnosis frequently missed or delayed, should be considered in any patient with right lower quadrant pain even if there is a history of appendectomy. Complete removal of the appendix is imperative and is the only mean to prevent the occurrence of this complication.

## Consent

Written informed consent was obtained from the patient for publication of this case report and accompanying images

## Competing interests

The authors declare that they have no competing interests.

## Authors' contributions

MG drafted and conceived the manuscript and FF SS RJ MH participated in its design
